# Reading deficits correlate with cortical and subcortical volume changes in a genetic migration disorder

**DOI:** 10.1097/MD.0000000000017070

**Published:** 2019-09-06

**Authors:** Wenyu Liu, Xintong Wu, Dong Zhou, Qiyong Gong

**Affiliations:** a Department of Neurology, West China Hospital; b Department of Radiology, Huaxi MR Research Center (HMRRC), West China Hospital, Sichuan University, Chengdu 610041, China.

**Keywords:** brain volume, neuronal migration disorder, periventricular nodular heterotopia, reading deficits

## Abstract

Periventricular nodular heterotopia (PNH) is the most common type of epileptogenic neuronal migration disorder, and often presents with epilepsy and reading disability. The functional role of ectopic nodules has been widely studied. However, the associated structural cortical and subcortical volumetric alterations have not been well characterized. Moreover, it is unknown whether a correlation between volumetric changes and behavioral problems exists.

40 subjects with bilateral PNH and 40 matched healthy controls were enrolled in this study. The total cerebral, gray matter, white matter, and cerebrospinal fluid (CSF) volumes were compared between the two groups. Furthermore, structural and functional correlations were evaluated between volumetric changes and reading disability.

There were no significant differences detected in total cerebral, gray matter or CSF volumes between the two groups, but there was a significant trend of larger gray-matter volume in PNH. Specifically, smaller white matter volumes were found in the PNH patients. Moreover, the volume of white matter was negatively related to time in the digit rapid naming task and a similar but insignificant trend was seen between the volume of gray matter and backward digit span.

These findings suggest that reading disability exists in our sample of bilateral PNH. Periventricular nodules would have normally migrated to the overlying cortex. However, the total cerebral, gray matter, and CSF volumes were unaffected. Alterations in neuronal migration may have an impact in the white matter associated reading dysfluency, that is, visually normal.

## 1 Introduction

Periventricular nodular heterotopia (PNH) is the most common type of gray matter heterotopia (GMH).^[1]^ Human cerebral cortex development is an incredibly complex and dynamic process.^[2,3]^ Neuronal migration produces heterotopia and is one of the most important steps during cortical development. In patients with PNH, clusters of normal neurons fail to migrate from the subependymal regions to the overlying cortex during cortical development.^[4]^ Though PNH is rare, as one of the most common cortical malformations, it is highly epileptogenic and is always associated with epilepsy. Bilateral PNH is the most common type of cortical malformation and presents as bilateral nodules along the ventricles.^[5]^ Bilateral PNH often has a genetic tendency caused by mutations in the *Filamin A* (*FLNA*) gene and is more prevalent in females.^[6,7]^

The nodules lining the ventricles in PNH result from faulty neuronal migration and can be detected by MRI. PNH can occur as an isolated cortical malformation or in association with other brain abnormalities.^[8]^ Nodules can vary in lateralization, number and size, but always line the ventricles. Despite these remarkable structural abnormalities, PNH has mainly been reported to be associated with epilepsy, mostly refractory epilepsy.^[9]^ Additionally, the specific behavioral abnormality related to PNH is difficulty reading though with normal intelligence.^[10]^ The main behavioral disability related to PNH is reading fluency, which is commonly known to be affected in neuronal migration disorders.^[11]^

Several lines of evidence have added to our knowledge of PNH. As a frequent developmental cause of drug-resistant epilepsy,^[12]^ heterotopic nodules are usually epileptogenic and structurally or functionally connected to the cortex, particularly the overlying cortex.^[13]^ This explains why patients with this disorder are resistant to a surgical treatment approach.^[14,15]^ When undergoing presurgical evaluation, the nodules are often surgically resected. However, after the resection, PNH patients often still have consistent seizures. Though the nodules are discrete, they are functionally integrated within the cortex.^[9]^ Complex associations exist between the nodules and the cortex, along with dysfunction affecting specific brain regions.^[16,17]^ Despite efforts to understand PNH, specific structural neuroimaging studies on PNH are insufficient. The cortical and subcortical organization of PNH is not well characterized, since most studies have focused on white matter or functional connectivity abnormalities.^[18,19]^ Because of the heterogeneous radiological features of PNH, previous studies were usually limited by small sample size and lacked systemic analysis.^[20–22]^ Moreover, how the structural changes correlate to the behavioral abnormalities remains unknown.

Therefore, this study focused on the cortical and subcortical volumetric changes in PNH compared to matched controls. Using a large sample of PNH patients, we utilized means of voxel-based morphometry to identify, quantify, and determine the distribution of cortical and subcortical abnormalities. Additionally, associations between structural changes and behavioral abnormalities were probed.

## 2 Methods

### 2.1 Subject criteria

This study was approved by local ethical committee of West China Hospital of Sichuan University. Written informed consent was obtained from each participant before the research procedure was initiated. We enrolled subjects with PNH from our epilepsy center in the Neurology department of West China hospital. Our inclusion criteria were as follows:

1. the presence of periventricular bilateral nodular heterotopia;
2. presence of seizures.

We excluded those that met any of the following conditions:

1. brain malformation on conventional T1 or T2-weighted MR images beyond PNH,
2. prior history of brain surgery,
3. alcohol/drug abuse or
4. history of any other neurological or psychiatric disease except epilepsy.

Additionally, for group comparison, age and sex-matched volunteers from the same regional population were recruited to serve as healthy controls (HC) through posters. The exclusions for HCs were as follows:

1. neurologic or psychiatric disorders,
2. apparent abnormalities on brain MRI including malformations of cortical development, cerebellar hypoplasia, polymicrogyria, microcephaly, and hydrocephalus, etc or
3. relation to one of the enrolled patients with PNH.

Moreover, the detailed demographic and clinical information including epilepsy duration, seizure frequency and response to treatment were all collected; patients were monitored every three months at the epilepsy center. Genetic tests had been applied in all of the patients with bilateral PNH and epilepsy, the detailed methods for genetic testing had been described in our previous work.^[23]^ An examination of mental state was first measured by Mini-mental State Examination (MMSE).

### 2.2 Data acquisition

A 3.0 T MRI scanner (Siemens Trio) with an 8 channel phase array head coil was applied in this study. Each subject used earplugs to reduce scanner noise. Structural MR imaging data were acquired with a standard protocol in PNH patients and the HCs. High resolution 3D-T1 images were acquired in axial orientation (TR: 1900 ms, TE: 2.26 ms, FOV: 256 mm × 256 mm, matrix: 512 × 512); the voxel size was 1 mm × 1 mm × 1 mm and slice thickness was 1 mm, flip angle = 90°. The number of slices was 176 and the total scan time is 427 s. During data collection, all subjects were instructed to relax, without falling asleep during scanning.

### 2.3 Heterotopic nodules volume analysis

**Figure 1:**
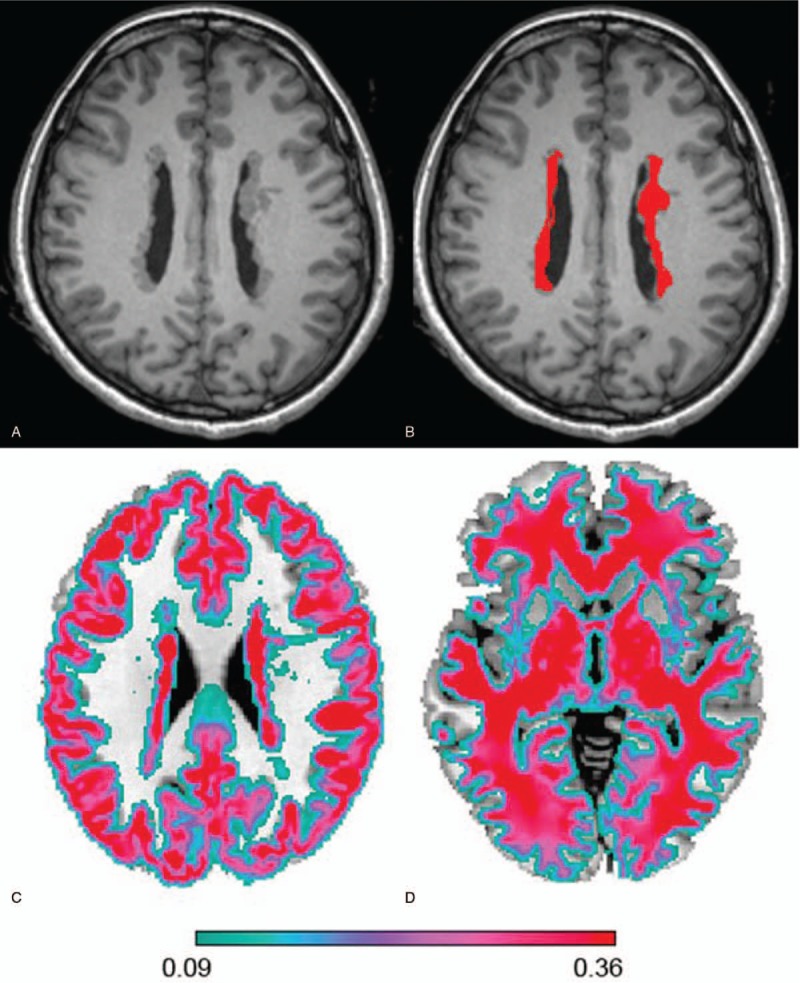
High resolution 3D-T1 imaging in a patient with bilateral PNH. (A) Axial image of PNH, showing bilateral periventricular nodular heterotopia. (B) The bilateral nodules were identified and outlined manually as regions of interest (ROIs). (C) Gray matter is segmented and outlined. (D) White matter is segmented and outlined.

Each individual nodule or cluster of nodules was identified and outlined manually as regions of interest (ROIs), slice by slice in the axial plane of 3D T1-weighted images (Fig. 1). The method of calculating volumes of the heterotopia ROIs has been described in our previous work.

### 2.4 Computational anatomy analysis

**Figure 2:**
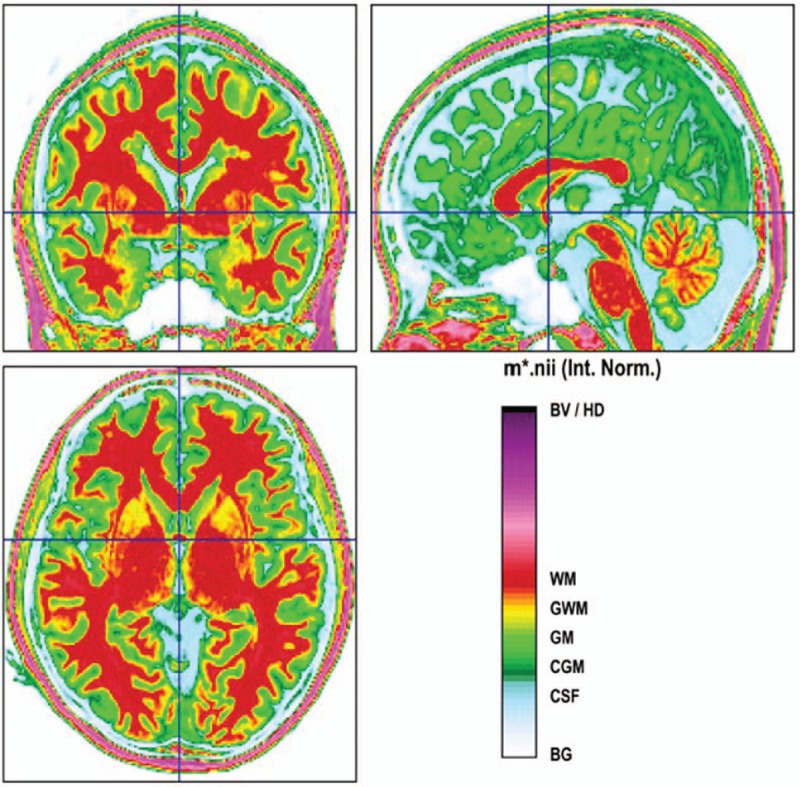
Structural component segmentations in patients with PNH and epilepsy using CAT software.

Computational anatomy toolbox (CAT12, http://dbm.neuro.uni-jena.de/cat/) analysis was performed by a toolbox of SPM12 (http://www.fil.ion.ucl.ac.uk/spm/), running on Matlab 2015a (Mathworks, Natick, MA, USA). Structural images were bias-corrected, tissue classified, and normalized to standard template using high-dimensional DARTEL normalization. Non-brain regions were removed including scalp and skull. Segmented images were normalized in MNI space and smoothed with an 8 mm Gaussian filter. Gray-matter volume was calculated modulating the normalized segmented images with a non-linear only warping resulting in an analysis of relative differences in regional gray-matter (Fig. 2).

### 2.5 Reading tests

#### 2.5.1 Rapid naming task (RAN)

Rapid naming tasks including digits, letters, and colors were performed in a quiet environment for all subjects. Before beginning these tasks, subjects were required to name digits/letters/colors in a practice trial to ensure familiarity.

Digit naming task. Five simple digits (3, 5, 6, 8, and 9) were repeatedly presented randomly, with eight rows and five digits in each row. Participants read out each digit in sequence as quickly as possible and were asked to complete the test twice, on two separate pages. The score was calculated by averaging and converting the total time for each participant.

Letter naming task. Five letters (b, l, g, r, and o) were repeatedly presented randomly. This task required each participant to read out the letters quickly, with six rows and five letters in each row. Scores were calculated as described above.

Color naming task. Five colors (blue, black, green, red, and yellow) were presented randomly. Participants read out the colors as quickly as possible the names, with five rows and eight colors in each row.

#### 2.5.2 Phonological and working memory

This test included both forward and backward digit span, and a character repetition task. The tasks were terminated after two consecutive failures.

Forward and backward digit span: The test included 18 sequences in total on a printed page, starting with three digits increasing one by one to ten digits in length maximally. Subjects repeated strings of digits in a positive sequence, in the same order as presented orally by the researcher. There were two sequences with the same length but different digits in each string. Afterwards, participants were instructed to read them in reverse of how they were presented.

For the character repetition task, subjects repeated strings of Chinese characters in the same order they were presented by the researcher, with 18 strings of characters in total beginning with two characters in a string increasing by one to nine characters in a string maximally. Two test items were used for each string length. Subject's performance scores were calculated as the number of character strings accurately repeated.

### 2.6 Statistical analysis

We used unpaired Student's *t* tests for examining differences regarding the demographic characteristics, clinical features, and reading performance between participants with PNH and control subjects using SPSS 20 software (SPSS, Chicago, IL, USA) (IBM, Armonk, New York, USA).

Differences of cortical and subcortical volumes were evaluated between the PNH group and HC group (InStat 3.10, GraphPad Software, Inc., San Diego, CA). Additionally, two-tailed Pearson correlation analyses were applied to assess the relationship between reading task performance and cortical and subcortical volumes. *P* < .05 was used for analysis.

## 3 Results

### 3.1 Subjects characteristics

The baseline features of the subjects are displayed in Table 1. A total of forty right-handed adult patients with PNH and epilepsy (23 females) were included who were previously diagnosed with bilateral PNH by MRI. They were all unrelated individuals. The *FLNA* gene analysis showed that 9 patients of the PNH group with bilateral nodules had *FLNA* mutations. In the PNH group the mean age was 25.1 years, and the standard deviation (SD) was 6.2 years (range 16–38 years). The mean onset age of seizures was 17.6 years and the SD was 5.7 years. After clinically presenting with seizures, all patients were diagnosed with PNH by MRI scan before being involved in this research. Based on the MMSE scores, none of the patients exhibited cognitive impairment.

**Table 1:**
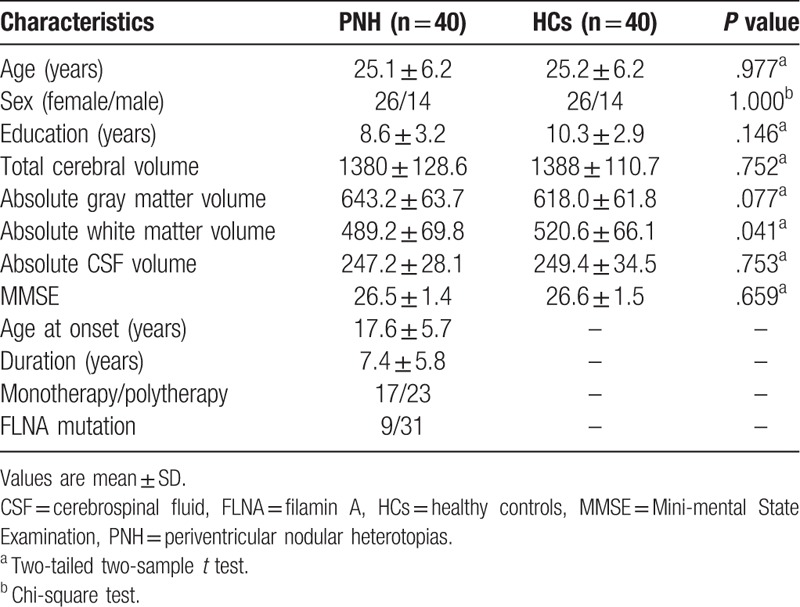
Basic information of patients with PNH and HCs.

Forty age and sex-matched HCs with negative MRI examinations were included for comparison. The mean age for the HCs group was 25.2 years (SD: 6.2 years). The mean value of education years in the control group was 10.3 years (SD: 2.9 years). There were no significant differences in age, sex, education years, handedness, or MMSE scores between the patient group and healthy group (*P* > .05).

### 3.2 Structural analysis

#### 3.2.1 Cortical and subcortical anatomical volume

**Figure 3:**
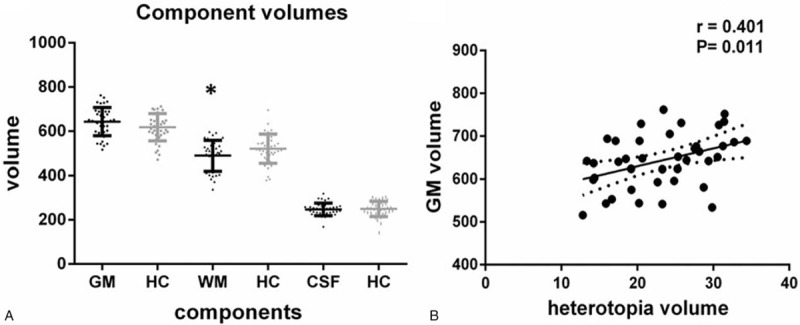
Component absolute volumes in PNH patients compared to controls. (A) The volume of white matter in the PNH group is significantly smaller than the controls. ^∗^*P* < .05. No significant differences in gray matter volume and CSF volume between two groups. (B) The volume of heterotopia is positively correlated with total gray matter volume. GM = gray matter, HC = healthy controls, WM = white matter.

The mean total cerebral volume in the PNH and HC group were 1380 mL (SD: 128.6) and 1388 mL (SD: 110.7), respectively. There was no significant difference detected between the groups (*P* = .75). However, a smaller volume of white matter (mean ± SD: 489.2 ± 69.8 vs 520.6 ± 66.1, *P* = .041) was seen in the PNH group. Additionally, a trend of greater gray-matter volume (mean ± SD: 643.2 ± 63.7 vs 618.0 ± 61.8, *P* = .077) was found in the PNH group compared to the HCs. However, there were no significant differences detected regarding CSF volume (247.2 ± 28.1 vs 249.4 ± 34.5, *P* = .75) (Fig. 3A).

The heterotopia volume in the PNH group occupied 1.68% of the total cerebral volume, and 3.59% of the total gray-matter volume. A significant positive correlation was found between heterotopia volume and the total gray-matter volume (r = 0.41, *P* = .01) (Fig. 3B).

#### 3.2.2 Effect of *FLNA* mutation on anatomical volume in PNH

Based on previous research, it appears that all the *FLNA*-mutated cases had bilateral lesions, whereas some of the non-*FLNA*-mutated cases had unilateral lesions; it is possible that a differential genetic influence be hypothesized due to different location of nodules. Though all the included patients were bilateral PNH patients, we classified the patients into *FLNA*-mutation group and non-*FLNA*-mutated group according to the genetic findings, and the results showed that there was no significant difference in anatomical volume between *FLNA*-mutated and non-*FLNA*-mutated groups (Table 2).

**Table 2:**
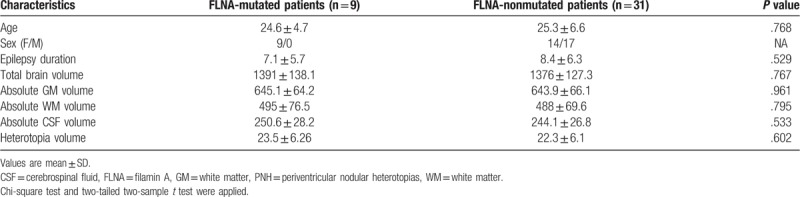
Difference between FLNA-mutated and FLNA-nonmutated patients with PNH.

### 3.3 Reading tests

The rapid naming (RAN) tasks included digit, letter, and color tests. For each test of RAN tasks, the average score was measured as the total time required for naming all subsets twice and the results were averaged to produce a score. The PNH group scored as follows; digit naming: 16.78 (SD: 2.68; median: 16; range: 11–25), letter naming: 20.73 (SD: 4.73; median: 20; range: 13–32), color naming: 26.51 (SD: 3.03; median: 16; range: 16–32). Phonological and working memory tasks were measured based on performance on three tests. The backward digit span test was related to verbal working memory. Forward digit span and character repetition tests were used to assess phonological memory. The mean forward digit span for PNH patients was 13.28 (SD: 2.58); the mean backward digit span was 7.88 (SD: 1.79) and the mean of Chinese character repetition span was 9.18 (SD: 1.72) (Table 3).

**Table 3:**
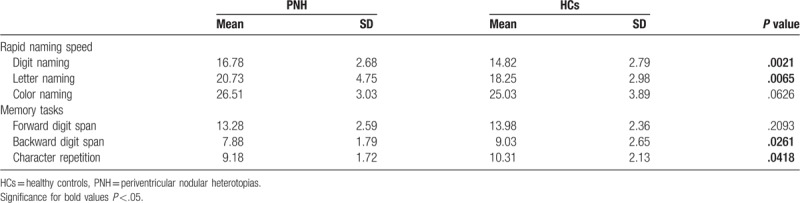
Different performance between two groups for reading tasks.

### 3.4 Reading skills correlates with altered brain volume

**Figure 4:**
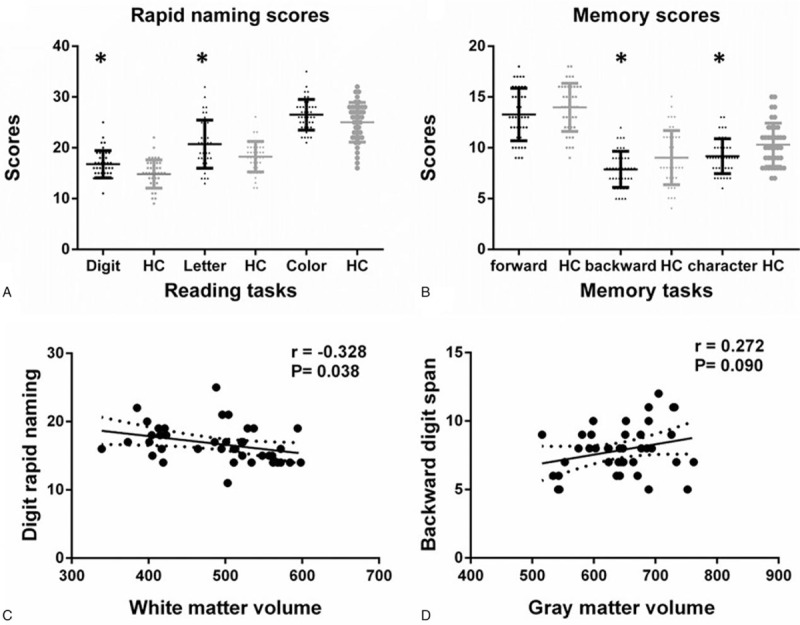
Reading tasks performance of the two groups and correlations to volumetric changes. (A) Rapid naming performances including digit, letter, and color rapid naming tasks were applied between the two groups. Significant difference was detected for digit naming. (B) Phonological and working memory tasks included forward, backward digit span, and character repetition tests. A trend but no significant difference was found in character repetition between the two groups. (C) The volume of white matter is negatively correlated to time for the digit rapid naming task (r = −0.33, *P* = .039). (D) A similar but not significant trend is present between volume of gray matter and backward digit span (r = 0.27, *P* = .092). GM = gray matter, HC = healthy controls, WM = white matter.

The correlations between Chinese reading performance and altered brain volume were evaluated and shown in Figure 4. The total cerebral volume had no significant correlation with any reading task performance tests. The volume of white matter was negatively correlated to time of the digit rapid naming task (r = −0.33, *P* = .039), but no correlation was detected with the other two RAN tasks. Moreover, a similar but insignificant trend was seen between gray-matter volume and backward digit span (r = 0.27, *P* = .092). The cortical and subcortical region volumes of the PNH group were not correlated with the other reading tests.

## 4 Discussion and conclusion

In this sample of PNH patients and epilepsy, we evaluated structural cortical and subcortical volume alterations including the gray matter, white matter, and CSF volume alterations in comparison to HCs and further correlated the volume alterations to reading task performance.

While the nodules vary in size, lateralization, location, and number, the imaging manifestations of PNH are remarkable. This study included bilateral PNH patients, aiming at evaluating the volumetric changes and association between heterotopia volume and total gray-matter volume. PNH results from aberrant migration producing nodules that were destined to migrate from subependymal regions toward the overlying cortex during cortical development.^[24]^ Neurons in the nodular heterotopia have normal morphology as revealed by multiple lines of molecular, pathological and immunohistochemical evidence.^[25–27]^ Their intended destinations are thought to be the overlying cortex to which they should have radially migrated.^[28]^

In the present study, patients with bilateral PNH and epilepsy had significantly smaller volumes of white matter, but no significant alteration in total cerebral, gray matter, or CSF volumes. Additionally, in PNH patients, larger gray-matter nodule volumes positively correlated with larger total cerebral volumes. This can explain the normal intelligence in subjects with PNH and epilepsy.^[29]^ However, a previous study suggested that the total cerebral volume, gray matter, white matter, or CSF volumes were not altered significantly in PNH subjects.^[22]^ The discrepancy between the two studies may be attributed to the enrolled sample of PNH patients. We enrolled only bilateral PNH patients that had at least four nodules lining the ventricles, and the volume of heterotopia was larger than those in the previous study which required only one nodule for enrollment. Therefore, the volume of white matter may be contracted as the heterotopic gray-matter nodules.

Despite the imaging findings, patients with PNH are always reported to have normal intelligence, and the most common behavioral problem in this population is reading disabilities. In the present study, we correlated cortical and subcortical volumes and heterotopia with this specific behavioral problem. We used the neuronal migration disorder, PNH, a developmental cortical malformation characterized by arrest in migration to explore Chinese reading disability. The results show that patients with PNH are deficient in rapid naming. Moreover, working memory is slightly impaired in this sample population. Altered diffusion metrics in the white matter organization and the white-matter pathways, including the arcuate fasciculus and corona radiate seem to play a pivotal role in dyslexia patients,^[30,31]^ while others attribute it to the corpus callosum.^[32]^ Consistent with previous studies, we found that decreased volume of white matter in PNH patients correlated with impaired digit rapid naming, emphasizing the key role of white matter in reading dysfluency in neuronal migration disorders. These novel results manifest the association between Chinese reading and white matter volume in patients with PNH, providing a potential mechanism for this specific cognitive disability in this sample population.

The epileptogenesis in PNH is attributed to a complete and widespread network including gray matter nodular heterotopia and particularly the overlying cortex. This was mainly reported in case reports using noninvasive functional MRI (fMRI),^[33,34]^ or EEG-fMRI.^[35]^ Recently, stereoelectroencephalography (SEEG) studies showed direct evidence revealing that even normal appearing cortexes had higher degrees of epileptogenicity.^[18,36]^ There is structural and functional connectivity between the nodules and cortex. The overlying cortex has the highest degree of connectivity; consistent with the notion that heterotopic gray matter would normally have radially migrated to the overlying cortex. These characteristics also explain the surgical failures in this disorder, particularly in bilateral PNH. Therefore, surgical techniques like stereotactic MRI-guided radiofrequency ablation or neuromodulation therapy may be effective approaches.^[24,37]^

The epileptogenic network in bilateral PNH is complex and widespread, and may be responsible for seizure generation in bilateral PNH. Researchers have mainly focused on the functional connectivity between the cortex and heterotopic nodules. However, quantitative anatomical measures of the heterotopic burden of PNH may help us to combine structural and functional associations in neuronal migration disorders, relating the volumetric alterations in this epileptogenic disorder to reading dysfluency and even cognitive disabilities.

Though all PNH patients included in this study had suffered epilepsy, most patients with PNH displayed age-appropriate development of their psychomotor and cognitive skills as well as completion of normal and professional education. A comparison of the demographic factors showed that compared to HCs, patients with PNH did not tend to be less educated. The normal range of cognitive and neurological findings in these patients is likely one reason for this, but perhaps more importantly, the average age of seizure onset in PNH patients is during early adolescence, which is relatively late compared to other epileptic patients and most likely means the majority of their education is complete before symptoms appear.

Additionally, structural abnormalities identified in ectopic nodules may be also associated with alterations in the vascular organization, therefore, impairment of cerebrovascular reactivity represent significant determinants of cognitive dysfunctioning.^[38]^ This can be an additional area to investigate in order to understand the pathophysiological mechanisms underlying the neuropsychological deficits in patients with PNH.

There are several limitations in our work. First, this study only included adult bilateral PNH patients. Future investigations with more types of PNH subjects and intelligence scores would be necessary to verify our results. Second, since all the subjects were treated with antiepileptic drugs, we could not rule out possible effects of medication. Third, all patients included were adults. However, longitudinal studies following patients from childhood into adulthood will be more valuable. Moreover, though we included bilateral PNH subjects, we did not report region-specific brain volumes regarding the lobe where the heterotopic nodules were located. Finally, in further research we will probe the cortical thickness in addition to the brain volume. Combining volume and morphology data will provide more information regarding diseases caused by arrest in neuronal migration.

## Acknowledgments

We would like to thank Prof. Wei Liao for his guidance regarding our data and statistic analysis and all the subjects took part in this study. This study was supported by National Natural Science Foundation of China (Grant No. 81420108014 and 81301186) and Post-doctor Research Project, West China Hospital, Sichuan University (Grant No. 2018HXBH075).

## Author contributions

**Conceptualization:** Xintong Wu, Dong Zhou.

**Funding acquisition:** Dong Zhou.

**Investigation:** Wenyu Liu.

**Methodology:** Wenyu Liu, Xintong Wu.

**Resources:** Qiyong Gong.

**Software:** Xintong Wu.

**Supervision:** Qiyong Gong.

**Visualization:** Dong Zhou.

**Writing – original draft:** Wenyu Liu.

**Writing – review & editing:** Dong Zhou, Qiyong Gong.

Dong Zhou orcid: 0000-0001-7101-4125.
